# Inebilizumab reduces neuromyelitis optica spectrum disorder risk independent of *FCGR3A* polymorphism

**DOI:** 10.1002/acn3.51911

**Published:** 2023-10-07

**Authors:** Ho Jin Kim, Orhan Aktas, Kristina R. Patterson, Schaun Korff, Amy Kunchok, Jeffrey L. Bennett, Brian G. Weinshenker, Friedemann Paul, Hans‐Peter Hartung, Daniel Cimbora, Michael A. Smith, Nanette Mittereder, William A. Rees, Dewei She, Bruce A. C. Cree

**Affiliations:** ^1^ Department of Neurology Research Institute and Hospital of National Cancer Center Goyang South Korea; ^2^ Medical Faculty Heinrich Heine University Düsseldorf Düsseldorf Germany; ^3^ Horizon Therapeutics Illinois Deerfield USA; ^4^ Department of Neurology Mellen Center for Multiple Sclerosis, Cleveland Clinic Ohio Cleveland USA; ^5^ Department of Neurology, Programs in Neuroscience and Immunology University of Colorado School of Medicine, Anschutz Medical Campus Colorado Aurora USA; ^6^ Department of Neurology University of Virginia Virginia Charlottesville USA; ^7^ Experimental and Clinical Research Center, Max Delbrück Center for Molecular Medicine and Charité Universitätsmedizin Berlin, Corporate Member of Freie Universitat Berlin and Humboldt‐Universitat zu Berlin Berlin Germany; ^8^ Brain and Mind Centre University of Sydney New South Wales Sydney Australia; ^9^ Department of Neurology Medical University Vienna Vienna Austria; ^10^ Department of Neurology Palacky University in Olomouc Olomouc Czech Republic; ^11^ Department of Neurology, UCSF Weill Institute for Neurosciences University of California San Francisco California San Francisco USA

## Abstract

Inebilizumab, a humanized, glycoengineered, IgG1 monoclonal antibody that depletes CD19+ B‐cells, is approved to treat aquaporin 4 (AQP4) IgG‐seropositive neuromyelitis optica spectrum disorder (NMOSD). Inebilizumab is afucosylated and engineered for enhanced affinity to Fc receptor III‐A (*FCGR3A*) receptors on natural killer cells to maximize antibody‐dependent cellular cytotoxicity. Previously, the F allele polymorphism at amino acid 158 of the *FCGR3A* gene (F158) was shown to decrease IgG‐binding affinity and reduce rituximab (anti‐CD20) efficacy for NMOSD attack prevention. In contrast, our current findings from inebilizumab‐treated NMOSD patients indicate similar clinical outcomes between those with F158 and V158 allele genotypes.

## Introduction

Neuromyelitis optica spectrum disorder (NMOSD) is an autoimmune disease that causes multifocal central nervous system inflammation.^1^ These acute inflammatory attacks may result in blindness, paralysis, neuropathic pain, and respiratory failure. Recovery from these attacks is often incomplete, resulting in permanent disability.^1^, ^2^, ^3^, ^4^ The presence of serum immunoglobulin G (IgG) autoantibodies against aquaporin‐4 (AQP4), a water channel protein mainly expressed on astrocytes, is highly specific to NMOSD and is present in approximately 75–90% of cases.^5^, ^6^, ^7^, ^8^

B‐cell depletion with the anti‐CD20 monoclonal antibody rituximab is often empirically used for attack‐prevention treatment.^9^, ^10^ Anti‐CD19 and CD20 monoclonal antibodies are B‐cell‐specific markers, however, CD19 targets antibody‐secreting plasmablasts, some plasma cells, and pre‐B cells.^11^, ^12^ Importantly, CD19+ plasmablasts and plasma cells (PB/PC) are often elevated in patients with NMOSD, especially during an attack, suggesting that these B‐cell subsets may play an integral role in attacks.^13^, ^14^, ^15^

A polymorphism in the low‐affinity IgG Fc region receptor III‐A (*FCGR3A*) gene has been linked to reduced efficacy of monoclonal antibody treatments, such as rituximab, in several diseases including NMOSD.^16^, ^17^, ^18^, ^19^, ^20^, ^21^ The *FCGR3A* protein gene encodes a potent cytotoxicity receptor (CD16a) on natural killer (NK) cells, monocytes, and macrophages and plays an important role in antibody‐dependent cell‐mediated cytotoxicity (ADCC). A *FCGR3A* polymorphism at amino acid position 158 results in either a valine (V158) or phenylalanine (F158) in the second Ig‐like domain and alters binding to its principal ligands.^22^ The V allele has a high affinity for IgG while the F allele is associated with decreased IgG‐binding affinity.^23^ A retrospective study of rituximab‐treated patients with NMOSD found that those with FF had a 5.5 times greater risk of an NMOSD attack versus those with VV.^16^

Inebilizumab is a humanized, glycoengineered, anti‐CD19 monoclonal antibody approved for the treatment of NMOSD in adults who are seropositive for AQP4.^24^, ^25^ Inebilizumab was engineered as an afucosylated monoclonal antibody based on the observation that human IgG1 molecules, which lack a core fucose residue in the Fc region, have enhanced affinity for the *FCGR3A* receptors and amplified ADCC capability.^24^, ^26^, ^27^, ^28^, ^29^

Here, the relationship between the *FCGR3A* polymorphism, NMOSD disease activity, and the treatment response to inebilizumab is evaluated in N‐MOmentum trial participants.

## Methods

### Study design

This post hoc analysis included 142 participants (inebilizumab, *n* = 104; placebo, *n* = 38) who consented for polymorphism genotyping from the N‐MOmentum (NCT02200770) trial which was a double‐blind, randomized, placebo‐controlled, Phase 2/3 trial evaluating the efficacy and safety of inebilizumab in 230 adults with NMOSD.^30^ The trial comprised of a 28‐week randomized controlled period (RCP) in which participants were allocated (3:1) to receive intravenous inebilizumab 300 mg or placebo, followed by an optional open‐label period (OLP) where all participants received inebilizumab 300 mg every 26 weeks for ≥2 years.^30^ Detailed methods and key clinical findings of the N‐MOmentum trial were previously published in 2019.^30^ Both the study protocol and statistical analysis plan are available at ClinicalTrials.gov: https://clinicaltrials.gov/ct2/show/NCT02200770.

### Genotyping and FACS analysis

Polymorphism genotyping utilized whole blood samples collected at the baseline visit to isolate whole blood DNA. DNA fragments were generated using the following primers: *FCGR3A* Forward; CCT GGT GTT TAC ATT GAG TTC TCC, *FCGR3A* Reverse; CCA ACT CAA CTT CCC AGT GTG AT. Genotyping for the *FCGR3A* polymorphism was performed on these fragments via a TaqMan SNP Genotyping Assay (ThermoFisher Assay ID: C__25815666_10). Accuracy was determined by Sanger sequencing (Detailed in Supplementary Materials).

B‐cell levels in peripheral blood were measured utilizing an ultrasensitive fluorescence‐activated cell sorting (FACS) assay with a lower limit of quantitation of 0.2 cell/μL, which is below the reportable clinical range (<12 cells/μL) used by commercial laboratories.^31^ B‐lineage cells were counted using CD20 as a FACS marker as bound inebilizumab interferes with CD19 based FACS. Further details on the methodology and reagents used for flow cytometry previously were reported.^32^

PB/PC‐specific gene expression was assessed by quantitative reverse transcription polymerase chain reaction of blood RNA samples and based on expression analysis of four genes (*IGHA1*, *IGJ*, *IGKV4‐1*, and *TNFRSF17*) that are expressed predominantly by CD27++CD38++ PB/PC in blood.^33^

### Outcomes

*FCGR3A* genotypes were compared for disease severity prior to trial enrolment, and response to inebilizumab were assessed at timepoints up to ≥4 years. Outcomes analyzed included annualized attack rates (AAR), risk of relapse, and disability as assessed by Expanded Disability Status Scale (EDSS) scores.

### Statistical analysis

The statistical analyses were conducted in R4.1.3. Differences in gene expression were assessed using Fisher's exact test. Differences in longitudinal CD20+ B‐cell count and CD20+ PC/PB gene signature were assessed by Mann–Whitney *U*‐test. Differences in attack rates and EDSS worsening between genotypes were modeled over time using a negative binomial regression model.

### Ethics

All participants provided written, informed consent. Ethics committees or institutional review boards at each study site approved the protocol. The study was conducted in accordance with provisions of the International Conference on Harmonization Good Clinical Practice Guidelines and principles of the Declaration of Helsinki in its currently applicable version. Genetic analysis was included as part of the clinical trial and all participants included in this analysis consented to have their DNA genotyped. Further details about ethical considerations were published previously.^30^

## Results

### Baseline clinical characteristics

Of the 142 participants who consented for genotyping, 14 (10%) were homozygous VV, 60 (42%) were heterozygous VF, and 68 (48%) were homozygous FF (Table 1). At baseline, significant differences were not observed among the genotype groups for demographics, disease severity, and disease duration prior to inebilizumab therapy. Most were women with a similar age of symptom onset and seropositive for AQP4‐IgG (VV 86%, F allele 90%). Prior azathioprine (AZA) or mycophenolate mofetil (MMF) treatment was reported in 58% (*n* = 43) of participants with the V allele compared to 40% (*n* = 27) of participants with the FF genotype (*p* = 0.03, Table 1).

**Table 1:** Baseline characteristics by *FCRG3A* genotype distribution.

Variable	*FCGR3A*						
	VV versus VF versus FF genotypes				V Carriers versus FF genotype		
	VV	VF	FF	*p*‐value	V	FF	*p*‐value
Patients, No (%)	14 (10%)	60 (42%)	68 (48%)	ns	74 (52%)	68 (48%)	ns
Onset age, mean (SD), years	43 (10)	42 (13)	40 (13)	ns	42 (12)	40 (13)	ns
Enrolment age, mean (SD), years	44 (10)	44 (12)	42 (13)	ns	44 (11)	42 (13)	ns
Female, No (%)	12 (86%)	51 (85%)	61 (90%)	ns	63 (85%)	61 (90%)	ns
Race				ns			ns
White	8 (57%)	24 (40%)	35 (68%)		32 (43%)	35 (51%)	
Black or African American	1 (7%)	6 (10%)	5 (7%)		7 (9%)	5 (7%)	
Asian	2 (14%)	16 (27%)	9 (13%)		18 (24%)	9 (13%)	
Other	3 (21%)	14 (23%)	19 (28%)		17 (23%)	19 (28%)	
Ethnicity							
Hispanic/Latin American	3 (21%)	13 (22%)	22 (32%)	ns	16 (22%)	22 (32%)	ns
AQP4‐IgG+, No (%)	12 (86%)	54 (90%)	61 (90%)	ns	66 (89%)	61 (90%)	ns
Time from onset to inebilizumab therapy, mean (SD)	1.0 (1.7)	2.5 (3.1)	2.5 (4.0)	ns	2.2 (2.9)	2.5 (4.0)	ns
Median	0.4	1.2	0.9		1.0	0.9	
Range, months	0.1–6.7	0.1–16.9	0.1–22.2		0.1–16.9	0.1–22.2	
Prior mitoxantrone treatment, No (%)	0 (0%)	2 (3%)	0 (0%)	ns	2 (3%)	0 (0%)	ns
Prior AZA or MMF treatment, No (%)	6 (43%)	37 (62%)	27 (40%)	**0.04**	43 (58%)	27 (40%)	**0.03**
Prior IFN‐β treatment, No (%)	0 (14%)	4 (7%)	2 (3%)	ns	4 (5%)	2 (3%)	ns
Prior rituximab treatment, No (%)	1 (7%)	4 (7%)	5 (7%)	ns	5 (7%)	5 (7%)	ns

. Statistical significance assessed via Fisher's exact test; AQP4, aquaporin‐4; AZA, azathioprine; IFN‐β, interferon beta, MMF, mycophenolate mofetil; No, number; SD, standard deviation.

### Pre‐study disease severity

Historical AARs in participants with >4‐year disease duration from NMOSD onset to study enrolment were higher in V allele participants (*n* = 27) than in FF genotype participants (*n* = 25). The median (interquartile range [IQR]) for the V allele was 0.9 (0.2–2.3) compared to the FF genotype, 0.4 (0.1–3.5), *p* = 0.045 (Table 2). Changes in EDSS scores were also higher among V allele participants with >4‐year disease duration from NMOSD onset to study enrolment. The median EDSS progression (IQR) for the V allele participants was 0.5 (0.2–1.9) compared to the FF genotype, 0.4 (0.07–0.8), *p* = 0.01. No significant differences in pre‐study disease severity were observed with the overall and >2‐year disease duration data (Table 2).

**Table 2:** Pre‐study disease severity of AAR and EDSS distributed by *FCGR3A* genotype.

Pre‐study AAR	FCGR3A						
	VV versus VF versus FF genotypes				V Carriers versus FF genotype		
	VV	VF	FF	*p*‐value	V	FF	*p*‐value
Overall, *n*	14	60	68		74	68	
Mean (SEM)	1.4 (0.2)	1.31 (0.08)	1.1 (0.1)	ns	1.3 (0.1)	1.2 (0.1)	ns
Median [range]	1.8 [0.4–7.3]	1.2 [0.2–9.6]	1.3 [0.1–8.9]		1.3 [0.2–9.6]	1.3 [0.1–8.9]	
Disease duration > 2 yrs., *n*	6	38	38		44	38	
Mean (SEM)	1.1 (0.2)	1.0 (0.1)	0.9 (0.1)	ns	1.0 (0.1)	0.8 (0.7, 0.9)	ns
Median [range]	0.6 [0.4–1.9]	1.0 [0.2–2.3]	0.7 [0.1–3.5]		1.0 [0.2–2.3]	0.7 [0.1–3.5]	
Disease duration > 4 yrs, *n*	4	23	25		27	25	
Mean (SEM)	1.1 (0.3)	0.9 (0.1)	0.7 (0.1)	ns	1.0 (0.1)	0.7 (0.1)	**0.045**
Median [range]	0.5 [0.4–0.6]	1.1 [0.2–2.3]	0.4 [0.1–3.5]		0.9 [0.2–2.3]	0.4 [0.1–3.5]	

Pre‐study EDSS progression in participants/year							
	VV	VF	FF	*p*‐value	V	FF	*p*‐value
Overall, *n*	14	60	68		74	68	
Mean (SEM)	2.0 (0.2)	1.9 (0.1)	1.8 (0.1)	ns	1.7 (0.1)	1.8 (0.1)	ns
Median [range]	2.9 [0.3–14.6]	1.2 [0.1–5.2]	1.6 [0.0–40.1]		1.4 [0.0–15.2]	1.6 [0.0–40.1]	
Disease duration > 2 yrs., *n*	6	38	38		44	38	
Mean (SEM)	0.9 (0.2)	0.8 (0.1)	0.7 (0.1)	ns	0.8 (0.1)	0.7 (0.1)	ns
Median [range]	0.8 [0.3–3.0]	0.8 [0.0–2.5]	0.7 [0.1–3.0]		1.0 [0.2–2.3]	0.7 [0.1–3.0]	
Disease duration > 4 yrs., *n*	4	23	25		27	25	
Mean (SEM)	0.7 (0.2)	0.5 (0.1)	0.4 (0.1)	**0.03**	0.6 (0.1)	0.4 (0.1)	**0.01**
Median [range]	0.6 [0.3–0.9]	0.5 [0.2–1.9]	0.4 [0.1–0.8]		0.5 [0.2–1.9]	0.4 [0.1–0.8]	

EDSS progression in participants/year, Patient's EDSS score divided by disease duration, assuming the patient started at an EDSS of 0 at study entry. Statistical significance assessed via negative binomial regression models where the impact of *FCGR3A* was as a “dose‐dependent model.” A regression was analyzed against EDSS and AAR where VV is classified as “2,” VF as “1,” and “FF” as 0. The second model was a “dominance model,” where VV and VF were both “1”s and FF were “0” in the model.

AAR, annualized relapse rate; EDSS, Expanded Disability Status Scale; *n*, number of participants; pts, patients; SEM, standard error of the mean; yr(s), year(s).

### Effects of long‐term inebilizumab treatment

**Figure 1:**
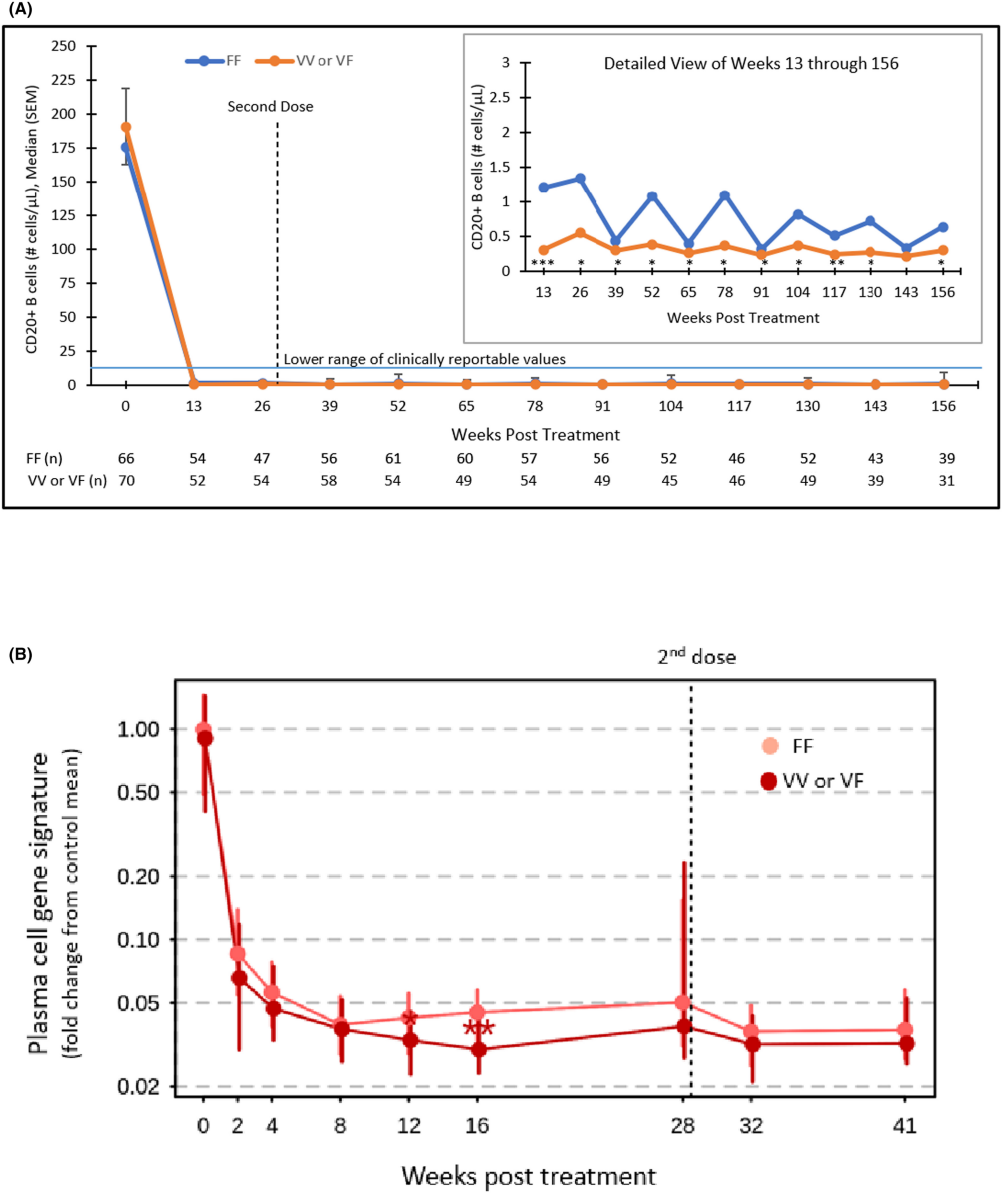
Effects of long‐term inebilizumab use on CD20+ B‐cell counts and plasma cell gene signature by FCGR3A genotype. (A) Longitudinal CD20+ B‐cell count by FCGR3A genotype. *p < 0.05, **p < 0.01, ***p < 0.001. Statistical significance was assessed using the Mann–Whitney *U*‐test. (B) Longitudinal CD20+ plasma cell gene signature by FCGR3A genotype. *p < 0.05; **p < 0.01. Statistical significance was assessed using Mann–Whitney *U*‐test.

There was little difference in the clinical metrics of NMOSD activity or B‐cell depletion between V allele and FF genotype participant subgroups. Depletion of CD20+ B‐cells was similar in V allele versus FF genotype participants at end of RCP and was sustained in both groups throughout the duration of the study (Fig. 1A). PB/PC gene signature represented as a median (IQR) fold‐change from the control mean showed a nominal difference at 12 and 16 weeks with end of the RCP results of 0.04 (0.02–0.20) in V allele participants (*n* = 52) versus 0.05 (0.03–0.20) in FF genotype participants (Fig. 1B).

There was no significant difference in risk of relapse (OR:0.94 (0.39, 2.24)) in recessive V allele carriers versus FF genotype participants. The AAR (standard error of the mean [SEM]) for participants on inebilizumab treatment were VV 0.00 (0.00), VF 0.10 (0.04), and FF 0.06 (0.03) over the duration of the study. EDSS worsening was also not different (OR:1.55 (0.54, 4.70)) in V allele carriers versus FF genotype participants over the study (Table 3).

**Table 3:** Clinical outcomes comparison of *FCGR3A* genotypes for the duration of N‐MOmentum.

*FCGR3A*	Relapse ≥ 1		Relapse ≥ 2		EDSS worsening
Variable	OR (95% CI)	*p*‐value	OR (95% CI)	*p*‐value	*p*‐value
VV versus VF versus FF Genotypes	1.29 (0.67, 2.64)	0.46	1.02 (0.28, 4.74)	0.98	0.98
Recessive V carriers versus FF genotypes	0.94 (0.39, 2.24)	0.89	0.68 (0.09, 4.21)	0.67	0.33
MAX		0.94		>0.99	>0.99

Statistical significance assessed by logic regression.

CI, confidence interval; EDSS, Expanded Disability Status Scale; MAX, maximum test statistic of additive and dominant models; OR, odds ratio.

## Discussion

This post hoc analysis evaluated 142 participants from the N‐MOmentum trial based on *FCGR3A* genotype and found that CD20+ B‐cell depletion was similar in V allele carriers versus FF genotype participants, and genotype did not appear to influence the AAR or EDSS worsening. The glycoengineered (afucosylated) Fc portion of inebilizumab could account for these findings as afucosylated monoclonal antibodies have up to a 10‐fold greater binding affinity to Fc receptor III‐A (*FCGR3A*) receptors on NK cells maximizing ADCC via IgG binding.^34^ In addition, a contributing factor to the lack of genotype bias may be the range of B‐cell depletion which occurs with the monoclonal CD19 antibody, including populations of antibody‐secreting plasmablasts, some plasma cells, and pre‐B cells.^11^, ^12^

In contrast, a retrospective review of 100 NMOSD patients treated with rituximab evaluating similar outcomes demonstrated that the FF genotype was associated with a 5.5 times increased risk of relapse compared to the VV genotype despite close B‐cell monitoring and redosing when CD19+ CD27+ B‐cells >0.05%.^16^ For this analysis, polymorphism genotyping in the N‐MOmentum study was only available for 10 participants with a history of prior rituximab treatment, limiting our ability to draw statistical conclusions from this sub‐group.

Diminished response to rituximab in patients with the FF genotype was also observed in several other autoimmune diseases including systemic lupus erythematosus and rheumatoid arthritis.^16^, ^17^, ^20^, ^21^, ^35^, ^36^, ^37^ Moreover, several studies demonstrated diminished response to rituximab for F carriers with follicular and non‐Hodgkin's lymphoma.^35^, ^38^ One study found that increasing the dose of rituximab in F carriers was a potential benefit; however, progression‐free survival remains lower in F carriers compared VV patients, even with markedly increased rituximab doses.^39^

Analysis limitations include that not all N‐MOmentum participants consented to genotype testing therefore, the number of individuals is a relatively small sub‐group for genotype–phenotype correlations. Further, the *post‐hoc* nature of this study limits conclusions to be exploratory rather than declarative, and significant *p*‐values should be interpreted as nominal. No head‐to‐head data are available to evaluate the efficacy of rituximab versus inebilizumab by FCGR3A polymorphism genotype. Future prospective studies will be necessary to determine whether inebilizumab has greater therapeutic benefit than rituximab in participants with the F allele of the *FCGR3A* polymorphism.

In conclusion, this analysis suggests that the beneficial effects of long‐term inebilizumab treatment offers consistent efficacy for patients regardless of *FCGR3A* polymorphism in NMOSD.

## Author Contributions

Ho Jin Kim, Orhan Aktas, Kristina R. Patterson, Schaun Korff, Michael A. Smith, Nanette Mittereder, and Bruce A. C. Cree were involved in the design/conceptualization of the study; acquisition, analysis/interpretation of data; and drafting/revising the manuscript for intellectual content. Amy Kunchok, Jeffrey L. Bennett, Brian G. Weinshenker, Friedemann Paul, Hans‐Peter Hartung, Daniel Cimbora, William A. Rees, and Dewei She were involved in analysis/interpretation of data and drafting/revising the manuscript for intellectual content.

## Funding Information

The N‐Momentum trial was funded by MedImmune/AstraZeneca and Viela Bio (now part of Horizon Therapeutics). Horizon Therapeutics supported the development of this manuscript and provided data analyses according to the direction of the authors.

## Conflict of Interest

Ho Jin Kim has received a grant from the National Research Foundation of Korea; consultancy/speaker fees or research support from Alexion, AprilBio, Altos Biologics, Biogen, Celltrion, Daewoong Pharmaceutical, Eisai, GC Pharma, HanAll Biopharma, Handok, Horizon Therapeutics (formerly Viela Bio), Kolon Life Science, MDimune, Merck Serono, Mitsubishi Tanabe Pharma, Novartis, Roche, Sanofi Genzyme, Teva‐Handok, and UCB; and is a co‐editor for the *Multiple Sclerosis Journal* and an associate editor for the *Journal of Clinical Neurology*.

Orhan Aktas reports grants from the German Research Foundation (DFG) and the German Ministry of Education and Research (BMBF); personal fees or research support from Alexion, Almirall, Bayer HealthCare, Biogen, Horizon Therapeutics (formerly Viela Bio), Merck Serono, Novartis, Roche, Sanofi, and Teva and has received travel reimbursement from the Guthy‐Jackson Charitable Foundation.

Amy Kunchok has received compensation for consultation and scientific advisory boards for Genentech, Horizon therapeutics, and EMD Serono.

Jeffrey L. Bennett reports payment for study design/consultation from MedImmune; personal fees from AbbVie, Alexion, Antigenomycs, BeiGene, Chugai, Clene Nanomedicine, Genentech, Genzyme, Reistone Bio, Roche, Imcyse, Mitsubishi Tanabe, and TG; grants from Alexion, and the National Institutes of Health. In addition, Dr Bennett has a patent “Compositions and methods for the treatment of neuromyelitis optica.”

Brian G. Weinshenker received payments for serving as chair of attack adjudication committees for clinical trials in NMOSD for Alexion, MedImmune, UCB Biosciences, and Horizon Therapeutics; has consulted with Chugai, Genentech, Horizon Therapeutics, Mitsubishi Tanabe Pharma, Roche Pharmaceuticals and CANbridge; and has a patent for NMO‐IgG for diagnosis of neuromyelitis optica, with royalties paid by Hospices Civils de Lyon, MVZ Labor PD Dr. Volkmann und Kollegen GbR, University of Oxford, and RSR. (updated 27 January 2022).

Friedemann Paul has received research support, speaker honoraria, and travel reimbursement from Bayer, Biogen Idec, Merck Serono, Novartis, Sanofi Genzyme, and Teva; is supported by the German Research Council (DFG Exc 257) and the German Competence Network for Multiple Sclerosis; has received travel reimbursement from the Guthy‐Jackson Charitable Foundation; and serves on the steering committee of the OCTIMS study, sponsored by Novartis.

Hans‐Peter Hartung has received fees for consulting, speaking, and serving on steering committees from Bayer HealthCare, Biogen Idec, Celgene Receptos, CSL Behring, GeNeuro, Genzyme, Horizon Therapeutics (formerly Viela Bio), MedDay, MedImmune, Merck Serono, Novartis, Roche, Sanofi, and TG Therapeutics with approval by the Rector of Heinrich Heine University Düsseldorf.

Kristina R. Patterson, Schaun Korff, Daniel Cimbora, Michael A. Smith, Nanette Mittereder, William A. Rees, and Dewei She are employees of Horizon Therapeutics and own stock.

Bruce A. C. Cree reports personal compensation for consulting from Alexion, Atara, Autobahn, Avotres, Biogen, Boston Pharma, EMD Serono, Gossamer Bio, Hexal/Sandoz, Horizon, Immunic AG, Neuron23, Novartis, Sanofi, Siemens, TG Therapeutics and Therini and received research support from Genentech.

## Supporting information


**Data S1** Supporting Information.
