# Supplementary material for: Inebilizumab reduces neuromyelitis optica spectrum disorder risk independent of FCGR3A polymorphism
Source: Ann Clin Transl Neurol. 2023 Oct 7;10(12):2413–20. doi: 10.1002/acn3.51911 (PMC10723240; doi:10.1002/acn3.51911)
Supplement: Supplementary file 1 — Data S1 Supporting Information. [file ACN3-10-2413-s001.docx]

**Supplementary Material**

**Title: Inebilizumab Reduces Neuromyelitis Optica Spectrum Disorder Risk Independent of *FCGR3A* Polymorphism**

**Author List:**

Ho Jin Kim, MD, PhD; ^1^ Orhan Aktas, MD; ^2^ Kristina R. Patterson, MD, PhD; ^3^ Schaun Korff, PhD, MSc, MS; ^3^ Amy Kunchok, MD;^4^ Jeffrey L. Bennett, MD, PhD;^5^ Brian G. Weinshenker, MD;^6^ Friedemann Paul, MD;^7^ Hans-Peter Hartung, MD, FRCP, FAAN, FANA, FEAN; ^8-11^ Daniel Cimbora, PhD;^3^ Michael A. Smith, PhD;^3^ Nanette Mittereder, MS;^3^ William A. Rees, MD;^3^ Dewei She, PhD;^3^ Bruce A.C. Cree, MD, PhD, MAS^12^

**Supplementary Methods**

Polymorphism genotyping utilized whole blood samples collected at the baseline visit to isolate whole blood DNA. 142 NMOSD participants who provided consent for genetic analysis were genotyped for the *FCGR3A* polymorphism via a TaqMan SNP Genotyping Assay (ThermoFisher Assay ID: C__25815666_10). Genotype calls were deemed high quality (> 99%) for 140 out of 142 samples in the initial analysis. Two samples with low quality calls were rerun a second time with resulting high quality genotype calls (>99%).

To further validate the reproducibility of the assay, 6 reference samples were run as 4 technical replicates with 100% agreement of genotype calls across all replicates for all samples. Finally, to examine accuracy of the TaqMan assay, Sanger sequencing was performed in parallel on a subset of 28 samples. For 24/28 samples, the Sanger sequencing result agreed with the TaqMan result. In the cases where the genotype call obtained from Sanger sequencing differed from that of the TaqMan assay, the Sanger result was carried forward for statistical analysis. No significant departure in allele frequencies from Hardy-Weinberg equilibrium was observed.
